# Suicide trends among Australian construction workers during years 2001–2019

**DOI:** 10.1038/s41598-022-24575-x

**Published:** 2022-11-23

**Authors:** Humaira Maheen, Yamna Taouk, Anthony D. LaMontagne, Matthew Spittal, Tania King

**Affiliations:** 1 Centre for Health Equity, Melbourne School of Population and Global Health, The University of Melbourne, Bouverie St, Carlton, 3010 Australia; 2 Institute for Health Transformation, School of Health and Social Development, Deakin University, Burwood, 3125 Australia; 3 Centre for Mental Health, Melbourne School of Population and Global Health, The University of Melbourne, Bouverie St, Carlton, 3010 Australia

**Keywords:** Epidemiology, Human behaviour

## Abstract

In many Western countries, including Australia, construction workers have been identified as being at elevated risk of suicide compared to other workers. A variety of suicide prevention initiatives have been implemented and expanded to reduce suicide in this occupational group; however, the net effect of these is unknown. Using 19 years of national suicide data, this study examined the suicide mortality of Australian male construction workers relative to all other working males, and compared suicide rates over time between the two groups. Age-standardized suicide rates were calculated for construction workers and those employed in other occupations. 2001–2019 trends in age-standardized rates of suicide mortality were analyzed by joinpoint regression analysis. The annual average percentage change (AAPC) measure was calculated for both groups to quantify change over time within each group, complemented by a pair-wise AAPC comparison of changes in trends between the two groups over the 2001–2019 period. Australian male construction workers' overall age-standardized suicide rate was 26.6 per 100,000 persons compared to 13.2 per 100,000 for male workers employed in other occupations (pooled over the entire 2001–2019 period). Over time, the suicide mortality rate declined in both construction workers and those working in other occupations; however, the decline in suicide mortality was greater in construction workers (AAPC: −3.0; 95%CI −4.0, −2.0) compared to other workers (AAPC: 1.5; 95%CI −2.1, −1.0). The AAPC pair-wise comparison showed a significant difference between the rate of decline among construction versus other workers over the 19-year study period (AAPC: −1.4; 95%CI 0.4, 2.5), confirming a rapid decline among construction versus other male workers. This study provides evidence of a decline in suicide rates among Australian construction workers over the last two decades. This decline may be attributable to the combined effects of population-wide, male-specific, and sector-specific suicide prevention efforts over this same period, suggesting that the continuation or expansion of such efforts may lead to further declines.

## Introduction

Excess mortality from suicide among construction workers has been observed in many Western countries^1–3^, including Australia^4,5^. In the United States, male workers in the construction and extraction industry (e.g., mining, natural gas, etc.) have the highest suicide rate of 49.4 per 100,000 among all occupational groups^6^. Such patterns were similarly noted in the UK (SMR 369, 95% CI 333–409), where the risk of suicide was higher in low-skilled construction workers compared to all other workers. Within the construction industry, differences in suicide rates have been noted between high vs low-skilled workers^4^ and males vs females^1^, where lower-skilled male construction workers (e.g., labourers) have been identified as being at the highest risk of suicide. This group also accounts for the majority of the construction workforce.

The etiology of suicide is complex and multifaceted, with a number of individual, occupational and contextual factors identified in previous research being important risk factors in construction workers. Firstly, construction workers are predominantly male—in Australia, recent labour force statistics identified that 88% of construction workers in Australia were men^7^—and men are at much greater risk of suicide than women both in Australia^8^ and in other Western countries^9^. Particular job-specific characteristics are also considered to increase suicide risk among construction workers, including limited job control^4,10^, job insecurity^5,10^, workplace bullying^10^, periods of unemployment or underemployment^5,10^, long working hours^5,10^, transient working conditions, travel and periods of time working away from family and support^5,10^. Other factors observed within the construction industry also thought to underpin suicide rates include relationship breakdown, personal debt, and excessive alcohol consumption, which may exacerbate these stressful conditions by diminishing social support networks and intensifying financial strain and stress^5,10^.

The construction sector accounts for a substantial proportion of the workforce internationally. In Australia, it employs around 10% of the Australian working population, with only retail and healthcare employing more people^7^. Construction is also a growing sector: over the period between 2001 and 2019, the number of people employed in the construction sector rose from more than 660,000 (7.7% of the Australian working population) to over 1.1 million (9.1% of the Australian working population)^7^. The above considerations have inspired sector-specific suicide prevention initiatives in construction over the last two decades^11–13^. Over the same period, other population-level initiatives have been implemented or expanded for all Australians, addressing mental health literacy, stigma reduction and help-seeking^14–16^, improving mental health services, and increasing pathways to help (e.g., toll-free help lines), and suicide prevention. Some of these population-level efforts have included male-tailored intervention content^17,18^. Taken together, the net effect of these intervention efforts could plausibly have an impact on suicide rates among construction workers. Thus, in this paper, our aim was to compare Australian national age-standardized male suicide rates among construction workers to rates in other working males over the period of 2001–2019 as a whole (pooled) and also assess and compare trends in these groups over the 2001–2019 period.

## Methods

### Study design

Drawing on coronial data from the National Coronial Information System (NCIS)^19^, this retrospective mortality study examined annual trends in suicide rates among male construction workers compared to workers in other occupations aged 15–64 years old and employed at the time of death.

### Ascertainment of cases

The NCIS online database offers a comprehensive search criterion (query builder program), enabling suicide cases to be extracted based on demographic information, year of death, and cause of death. For this study, only cases where the intent type at completion was intentional self-harm were included. Cases are categorized as 'open' or 'closed' based on the current status of the coronial investigation; only 'closed' cases were included for analysis because they have a complete coronial record. The analytic sample was restricted to employed cases with occupational information (see below).

### Ascertainment of occupations

**Figure 1:**
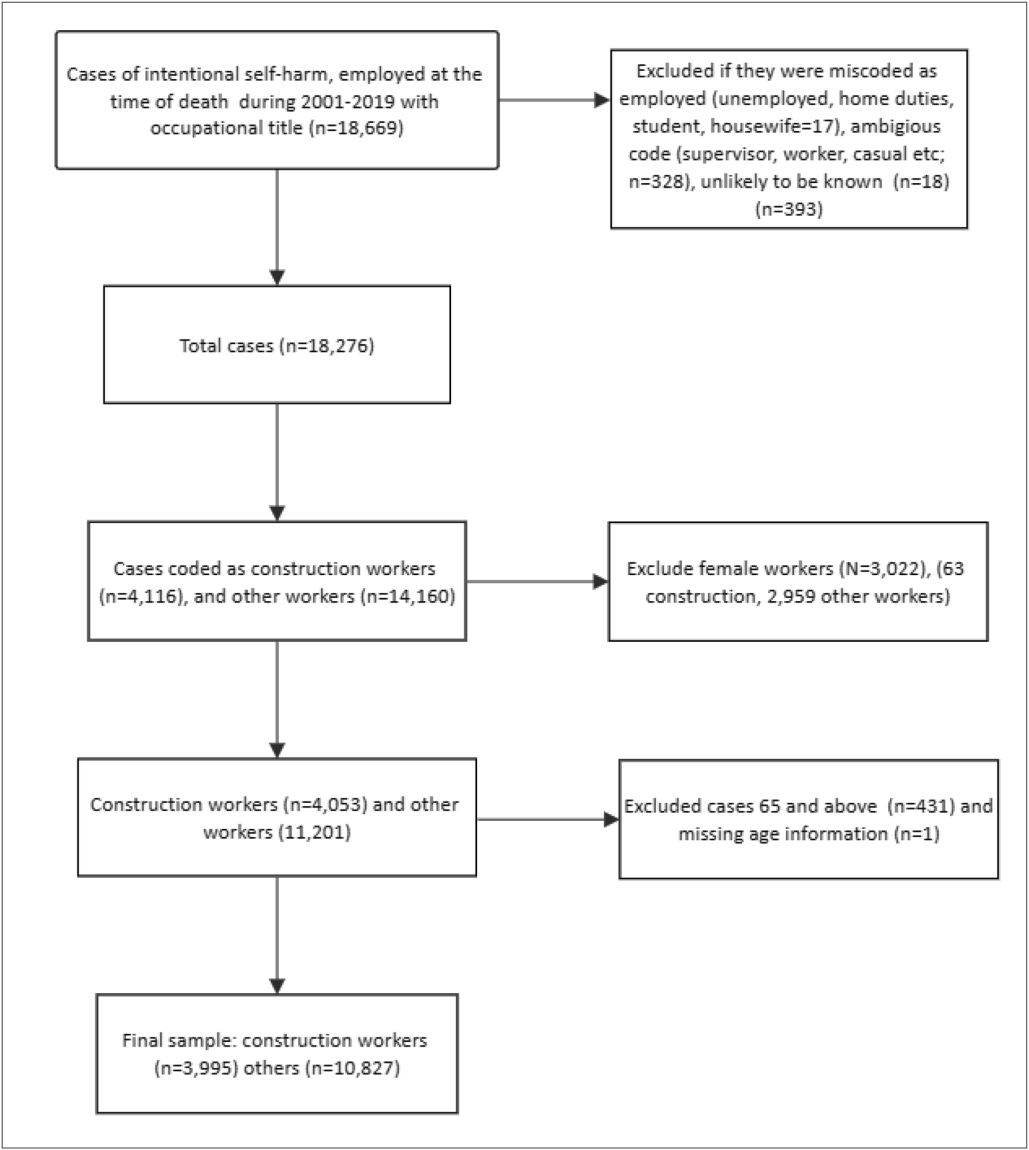
Flow chart for case selection.

The occupation of all suicide cases listed in the NCIS from 2001 to 2019 was ascertained using the Australian and New Zealand Standard Classification of Occupations (ANZSCO), coded to four digits^20^. Those in the construction industry were identified as being involved in the building of homes, dwellings, buildings, or other structures and roads, as per the ANZSCO classifications^20^. Construction work was also defined as building work related to additions, alterations, reconstruction, maintenance and repairs^20^. Applying this definition, occupation was coded into two groups, 'construction workers' and 'other workers'. Supplementary file 1 provides ANZSCO codes used in this study. Cases where employment status was misclassified (unemployed cases misclassified as employed) or where occupational information was either ambiguous or unavailable were dropped (2%). Further, we excluded females from the analysis because there were relatively few suicide deaths (1.6%) among females in the construction sector over the time period 2001–2019. Figure 1 shows the case selection process.

### Population estimates

Population-level sex, age, and occupational data at ANZSCO level 4 was obtained from the Australian Census information. Census data for the years 2006, 2011 and 2016 were used as population estimates. We used Census 2006 data as the reference year for suicide cases between 2001 and 2005, Census 2011 data as the reference year for suicide cases between 2006 and 2010, and Census 2016 as the reference year for suicide cases between 2011 and 2019. The construction industry has changed substantially over time, and to account for this, populations were adjusted using the quarterly released labour force data^7^. The adjustment accounts for the average change in the working population (each year) with reference to the corresponding census year. The Australian standard population (2001) from the ABS (ABS, 2015) was used to calculate age-standardized suicide rates.

### Analysis

Age-standardized suicide rates per 100,000 person-years were calculated using adjusted population data for two groups: (1) construction workers and; (2) workers in all other occupations. We used joinpoint regression to assess and compare long-term trends in suicide mortality rates of both groups.

Joinpoint regression fits a series of joined straight lines on a log scale to the trends in the rates, where each joinpoint represents a change in trend at a p < 0.05 level^21^. When comparing two independent groups, the analysis compares two-segmented regression lines (one for each group) to test whether the two lines are parallel, allowing different intercepts^22^. As a first step, the analysis identifies the best fitting model for each group. It does this by fitting a model with the minimum number of joinpoints (i.e., 0 joinpoints, which is a straight line) and then testing whether further joinpoints (up to a pre-defined number of joinpoints offer a significantly better fit to the data^21,22^. The final models are then used for comparing trends between the two groups.

The joinpoint analysis produces two measures of trends; (1) annual percentage change (APC) and (2) average annual percentage change (AAPC)^21^. The APC measures significant changes in trend corresponding to each time segment, whereas the AAPC provides a summary measure of a trend change for the whole period. The AAPC is a weighted average of the APCs, with the weights equal to the length of the joinpoint segments; hence it accounts for trend transitions (if there are any) during the study time. Unlike the conventional annual percentage change, which assumes linearity of rates over time, the AAPC assumes that the change in age-adjusted rates is constant over each time segment defined by the transition points but varies among different time segments^21^. If there are no significant trend transitions, the AAPC is approximated by the APC. When comparing trend changes in two groups over a similar period, the AAPC provides more meaningful results than the APC measure because the comparison is made on an equal length of data compared to APC, which may have different transition points for different groups. The joinpoint analysis also provides the AAPC pair-wise comparison measure to compare trend differences of groups over a similar period of time^21,22^.

We set our maximum number of joinpoints to three, which we regarded as reasonable given the data length of 19 years^22^. Based on the final model, we computed APC, AAPC, and AAPC pair-wise comparison measures. STATA SE 16.0 was used to calculate age-standardized suicide rates, and the joinpoint analysis was performed using the joinpoint software (Version 4.9.0.1) from the Surveillance Research Program of the US National Cancer Institute^23^.

### Ethics approval and consent to participate

The study was approved by the Victorian Justice Human Research Ethics Committee (reference CF/18/22468), Department of Justice and Community Safety and the Human Ethics Advisory Group, School of Population and Global Health, University of Melbourne. Given the retrospective nature of the study, where study participants were deceased, it was not possible to ask for informed consent; therefore, both the Victorian Justice Human Research Ethics Committee, Department of Justice and Community Safety and Human Ethics Advisory Group, School of Population and Global Health, University of Melbourne approved the waiver for informed consent. The authors assert that all procedures contributing to this work comply with the ethical standards of the relevant national and institutional committees on human experimentation and with the Helsinki Declaration of 1975, as revised in 2008.

## Results

During 2001 to 2019, there were 14,822 suicides among employed males aged 15–64 years with a known occupation. Of these, 3,995 suicides were among males employed as construction workers and 10,827 were among those employed in other jobs at the time of death. The mean age at the time of death for construction workers (37.9 years) was lower than other workers (40.8 years). Table 1 compares suicide mortality between construction workers and other workers at the time of death. Overall, the male construction workers' age-standardized suicide rate (ASR) was 26.6 per 100,000 persons; for other male workers, the ASR was 13.2 per 100,000 persons.

**Table 1:** Australian male suicide mortality comparison of construction vs other workers (2001–2019; pooled, all years).

	Construction workers	Other workers
Number of suicide	3995	10,827
ASR (per 100,000), 95% CI	26.6 (25.8, 27.4)	13.2 (12.9, 13.4)
AAPC, 95% CI, p-value	−3.0 (−4.0, −2.0; p < 0.001)	−1.5 (−2.1, −1.0; p < 0.001)
AAPC comparison, 95% CI, p-value	1.4 (0.4, 2.5; p < 0.001)	

**Figure 2:**
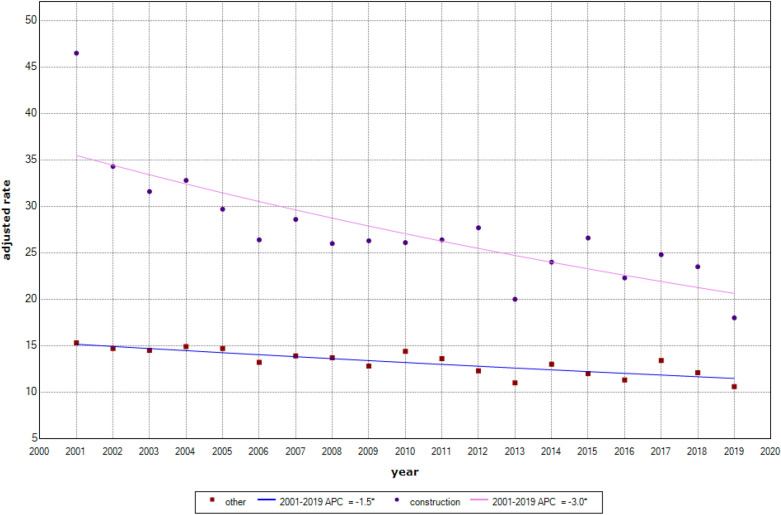
Comparision for annual percentage change in age-standardised suicide rates of construction vs other workers (final model: 0 joinpoint (s) for both groups).

The joinpoint algorithm identified that the best fitting model for each group was a model with 0 joinpoints (i.e. a linear trend); see Supplementary file 2. At the start of the study (year 2001), male construction workers had a substantially higher suicide rate than other workers (46.5 vs 15.3 per 100,000). The individual AAPC summary measures showed that suicide rates declined in both groups over the study period, with suicide rates among construction workers showing a 3.0% annual decrease, while among all other workers, there was a 1.5% annual decrease. The AAPC pair-wise comparison measure showed a statistically significant difference (AAPC 1.4, p < 0.001), confirming that, on average, the annual rate of change for construction workers was more rapid than for other workers over the 19 years of the study period. Figure 2 graphically presents the estimated suicide rates between the two groups.

### Sensitivity analysis

Given the high suicide rate of construction workers in 2001, we did a sensitivity analysis by excluding the 2001 data point but found no change in AAPC. Furthermore, we also calculated suicide rates using Australian and New Zealand Standard Industrial Classification (ANZSIC) code for the construction industry and found a minimal difference in the overall findings (ASR construction workers: 27.6, 95% CI 26.4–28.2) and (AAPC comparison measure 1.8, 95% CI 0.5–3.1).

## Discussion

This analysis of 19 years of national suicide mortality data provides evidence that the suicide rate among Australian employed males has declined and that the rate among construction workers has declined at a greater rate than other employed Australian males.

National age-standardized suicide rate trends for males have increased from 16.2 per 100,000 population in 2011 to 18.6 per 100,000 in 2020; however, these estimates include the entire male population, whether employed or not (ABS 2022). Given that employed individuals are known to have lower rates of suicide than unemployed persons, our results are plausibly consistent with these. That is, the overall higher rate (16.2—18.6, 2011–2020) observed in the whole population of males (that includes unemployed males as well as those employed in high-risk occupations) would be expected to be higher than that of employed males, excluding construction workers (average of 13.2 over 2001–2019), and lower than that for construction workers, who, despite being employed, have an average of 26.6/100,000 over the 2001–2019 period. In this light, our findings showing a decline in suicide rates for employed males overall and construction workers, particularly, are promising—limitations below notwithstanding.

### Study limitations

While the NCIS data provides the most accurate, comprehensive information regarding suicide deaths that is available in Australia, some limitations must be acknowledged. First, some of the occupations listed by the Coroner are ambiguous or insufficient for coding purposes. This results in 2% missing data, which is minimal loss in this study. It is also important to note that coronial processes can be lengthy, with some cases remaining 'open' for years. As a consequence, suicide cases may be under-counted in more recent years. There are also variations in coronial processes that exist across state and territory jurisdictions, which may contribute to some inconsistencies and undercounting. In particular, inconsistencies in the determination of intent may lead to substantial undercounting in some jurisdictions^24^. It is estimated that suicides are underestimated by 11–16% due to an inability to judge intent (such as in single-occupant car crashes)^24^. We also highlight the fact that we have categorized construction workers according to the ANZSCO occupations denoted in Supplementary file 1. Not all of these occupational codes distinguish between sectors, and the "construction worker" category is likely to include some workers employed in smaller sectors such as the mining and energy sectors, potentially leading to measurement error in estimates. Having said that, since construction is a fairly large industry, the misclassification of small number of mining and energy workers as construction workers is unlikely to affect results (refer sensitivity analysis).

We note that we compared suicide rates among construction workers to others in the employed population because mental illness and suicide rates are generally higher among the unemployed and those who are 'not in the labour force' (NILF). This is known as the 'healthy worker effect'^25^. Comparing rates in specific occupational groups to the general population would thus be compared to an elevated reference driven up by unemployment and NILF, thus biasing estimates of relative risk downwards for groups with relatively high rates in the working population. By using other employed workers, we have mitigated such bias.

### Implications of results

Assuming the observed trends are real, we can only speculate on what might explain them, as the present analysis does not direcltly address this question. Employed males might experience a decline in suicide rates compared to an increase among all males because they have a lower suicide risk in the first place (healthy worker effect) and have lower rates of mental illness, both of which will reduce suicide risk compared to those who are unemployed or Not in the Labour Force (NILF)^26^. Further, employed males may be better placed to benefit from expanded state and commonwealth suicide prevention programs^27^ that have demonstrated some impact on suicide behaviour in the general population and mental health services^28,29^.

Construction workers might experience greater declines in suicide rates due to other factors combined with the potential impacts described in the preceding paragraph. First, construction workers started from roughly two times the rate of other working males in the early 2000's, leaving greater room for improvement. That the observed trend is fairly progressive and consistently downward over 19 annual estimates suggests that this would be more than a regression to the mean or some other statistical aberration. The state of economic activity in the construction sector has shown substantial improvement during the study time^30^, and could have played some role in influencing suicide trends. Building ‘starts’ and ‘completions’ increased markedly from 2012 to 2019, up until the onset of the COVID-19 pandemic. When there is an increasing demand for building and construction labour, as implied by the increased activity in the sector, working conditions, including exposure to psychosocial job hazards such as job insecurity, are improved^31^; this in turn could reduce suicide risk for construction workers^32^. In the absence of readily available construction sector unemployment rates, we can also consider the national unemployment rates. In the early 2000s, unemployment was between 6 and 7% in Australia, and since the mid-2000s, it has varied between 4 and 6%^33^. Thus trends in both sector economic activity and unemployment rates may have contributed to declines in construction worker suicide.

There have also been considerable sector-specific suicide prevention initiatives in the Australian construction industry which may have contributed to the decline in suicide rates. Since the 2003 Cole Royal Commission report identified suicide as a possible reason for 41% of all deaths among Queensland building and construction workers^34^, the Australian construction industry has demonstrated a significant commitment to suicide prevention in its workforce^13,33^. Programs such as MATES in Construction^13^ and the similar Incolink Blue Hats program have implemented multimodal approaches to reduce stigma towards mental health, increase suicide prevention literacy and promote help-offering and help-seeking behaviour^12,13,35^. Other programs have targeted higher-risk workers within the sector, such as young apprentices from rural and regional areas^11,12^. Evaluation of these programs suggests reduced stigma, increased self-reported suicide awareness and a positive attitudinal change towards help-seeking^11,13,36–39^. However, these programs have operated on a state-by-state basis instead of nationally. MATES has had the greatest reach in Queensland and also operates in Western Australia, South Australia, New South Wales, and the Northern Territory. An estimated > 300,000 workers participated in MATES, and over 23,000 of those workers have volunteered to be trained as on-site 'Connectors,' their role being to connect workers in distress to help)^40^. Given the reach of construction-specific programs and targeted interventions, it is possible that it has contributed to suicide prevention among construction workers.

Despite the apparent decline in suicide rates among construction workers, they remain at elevated risk compared to other workers. Our findings suggest that the combined effects of all relevant intervention efforts—from the population level to the sector-specific level—may be having a positive impact. Continued efforts by the Australian construction industry to prioritize employee mental wellbeing, promote helping behaviours and seek ways to reduce work-related stressors could plausibly contribute to continuing declines in the suicide rate in the sector.

## Supplementary Information

Supplementary Information.

## Data Availability

The datasets analysed during the current study are not publicly available due to confidentiality reasons. The corresponding author can provide deidentified aggregated data as per the NCIS guidelines to someone who is authorised by the Victorian Justice Human Research Ethics Committee and NCIS for access to the data.
