# Supplementary material for: Suicide trends among Australian construction workers during years 2001–2019
Source: Sci Rep. 2022 Nov 23;12:20201. doi: 10.1038/s41598-022-24575-x (PMC9686251; doi:10.1038/s41598-022-24575-x)
Supplement: Supplementary file 1 — Supplementary Information. [file 41598_2022_24575_MOESM1_ESM.docx]

**Supplementary material 1 : Occupational Coding**

Occupational information for suicide cases was derived from the Australian and New Zealand Standard Classification of Occupations (ANZSCO) to the four-digit level. Those cases described as belonging to the construction industry are those occupations shown in red font below:

Construction workers

1331 Construction Managers

3121 Architectural, Building and Surveying Technicians

3122 Civil Engineering Draftspersons and Technicians

3123 Electrical Engineering Draftspersons and Technicians

3124 Electronic Engineering Draftspersons and Technicians

3125 Mechanical Engineering Draftspersons and Technicians

3126 Safety Inspectors

3129 Other Building and Engineering Technicians

3311 Bricklayers and Stonemasons

3312 Carpenters and Joiners

3321 Floor Finishers

3322 Painting Trades Workers

3331 Glaziers

3332 Plasterers

3333 Roof Tilers

3334 Wall and Floor Tilers

3341 Plumbers

3411 Electricians

3421 Airconditioning and Refrigeration Mechanics

3422 Electrical Distribution Trades Workers

3423 Electronics Trades Workers

3424 Telecommunications Trades Workers

7111 Clay, Concrete, Glass and Stone Processing

7112 Industrial Spray painters

7113 Paper and Wood Processing Machine Operators

7114 Photographic Developers and Printers

7115 Plastics and Rubber Production Machine Operators

7116 Sewing Machinists

7117 Textile and Footwear Production Machine Operators

7119 Other Machine Operators

7121 Crane, Hoist and Lift Operators

7122 Drillers, Miners and Shot Firers

7123 Engineering Production Systems Workers

7129 Other Stationary Plant Operators

8211 Building and Plumbing Labourers

8212 Concreters

8213 Fencers

8214 Insulation and Home Improvement Installers

8215 Paving and Surfacing Labourers

8216 Railway Track Workers

8217 Structural Steel Construction Workers

8219 Other Construction and Mining Labourer

All other occupations

Excluding the occupations listed above

**Supplementary materials 2**

Model selection method

| Cohort | Test Number | Null Hypothesis | Alternate Hypothesis | Numerator Degrees of Freedom | Denominator Degrees of Freedom | Number of Permutations | P-Value | Significance Level~ |
| --- | --- | --- | --- | --- | --- | --- | --- | --- |
| Other | #1 | 0 Joinpoint(s)* | 3 Joinpoint(s) | 6 | 11 | 4500 | 0.425 | 0.0167 |
| Other | #2 | 0 Joinpoint(s) * | 2 Joinpoint(s) | 4 | 13 | 4500 | 0.709 | 0.0167 |
| Other | #3 | 0 Joinpoint(s) * | 1 Joinpoint(s) | 2 | 15 | 4500 | 0.885 | 0.0167 |
| construction | #1 | 0 Joinpoint(s) * | 3 Joinpoint(s) | 6 | 11 | 4500 | 0.067 | 0.0167 |
| construction | #2 | 0 Joinpoint(s) * | 2 Joinpoint(s) | 4 | 13 | 4500 | 0.059 | 0.0167 |
| construction | #3 | 0 Joinpoint(s)* | 1 Joinpoint(s) | 2 | 15 | 4500 | 0.022 | 0.0167 |

* selected method

~ significance level calculated based on the permutation, for the individual test

Final selected model : other – 0 joinpoint (s), construction workers 1 (joinpoint)

**Test for paralleism**

| Kmax^ | Numerator Degrees of Freedom | Denominator Degrees of Freedom | Number of Permutations | P-Value | Significance Level~ |
| --- | --- | --- | --- | --- | --- |
| 0 Joinpoint(s) | 1 | 34 | 4500 | 0.007111 | 0.05 |

Final selected model (Reject parallelism)
